# Targeting succinate dehydrogenase with malonate ester prodrugs decreases renal ischemia reperfusion injury

**DOI:** 10.1016/j.redox.2020.101640

**Published:** 2020-07-12

**Authors:** Timothy E. Beach, Hiran A. Prag, Laura Pala, Angela Logan, Margaret M. Huang, Anja V. Gruszczyk, Jack L. Martin, Krishnaa Mahbubani, Mazin O. Hamed, Sarah A. Hosgood, Michael L. Nicholson, Andrew M. James, Richard C. Hartley, Michael P. Murphy, Kourosh Saeb-Parsy

**Affiliations:** a Department of Surgery and Cambridge NIHR Biomedical Research Centre, Biomedical Campus, University of Cambridge, Cambridge, CB2 0QQ, UK; b MRC Mitochondrial Biology Unit, University of Cambridge, Cambridge, CB2 0XY, UK; c School of Chemistry, University of Glasgow, Glasgow, G12 8QQ, UK

**Keywords:** Ischemia reperfusion injury, Malonate, Succinate, Mitochondria, Kidney, Succinate dehydrogenase, CAC, citric acid cycle, DMM, dimethyl malonate, DMSO, dimethyl sulfoxide, EVNP, *ex vivo* normothermic perfusion, FMN, flavin mononucleotide, IR injury, ischemia reperfusion injury, IVC, inferior vena cava, LN_2_, liquid nitrogen, MAM, diacetoxymethyl malonate, MnSOD, manganese superoxide dismutase, MS, mass spectrometry, RET, reverse electron transport, ROS, reactive oxygen species, SDH, succinate dehydrogenase

## Abstract

Renal ischemia reperfusion (IR) injury leads to significant patient morbidity and mortality, and its amelioration is an urgent unmet clinical need. Succinate accumulates during ischemia and its oxidation by the mitochondrial enzyme succinate dehydrogenase (SDH) drives the ROS production that underlies IR injury. Consequently, compounds that inhibit SDH may have therapeutic potential against renal IR injury. Among these, the competitive SDH inhibitor malonate, administered as a cell-permeable malonate ester prodrug, has shown promise in models of cardiac IR injury, but the efficacy of malonate ester prodrugs against renal IR injury have not been investigated. Here we show that succinate accumulates during ischemia in mouse, pig and human models of renal IR injury, and that its rapid oxidation by SDH upon reperfusion drives IR injury. We then show that the malonate ester prodrug, dimethyl malonate (DMM), can ameliorate renal IR injury when administered at reperfusion but not prior to ischemia in the mouse. Finally, we show that another malonate ester prodrug, diacetoxymethyl malonate (MAM), is more potent than DMM because of its faster esterase hydrolysis. Our data show that the mitochondrial mechanisms of renal IR injury are conserved in the mouse, pig and human and that inhibition of SDH by ‘tuned’ malonate ester prodrugs, such as MAM, is a promising therapeutic strategy in the treatment of clinical renal IR injury.

## Highlights

• Accumulation of succinate during renal ischemia is conserved across species.
• Succinate dehydrogenase is a key therapeutic target in renal ischemia-reperfusion.
• Malonate may inhibit succinate dehydrogenase during ischemia and on reperfusion.
• Ester prodrugs enable delivery of malonate to succinate dehydrogenase *in vivo*.
• Malonate ester prodrugs may be ‘fine-tuned’ to optimise their delivery and efficacy.

## Graphical abstract

**Figure:**
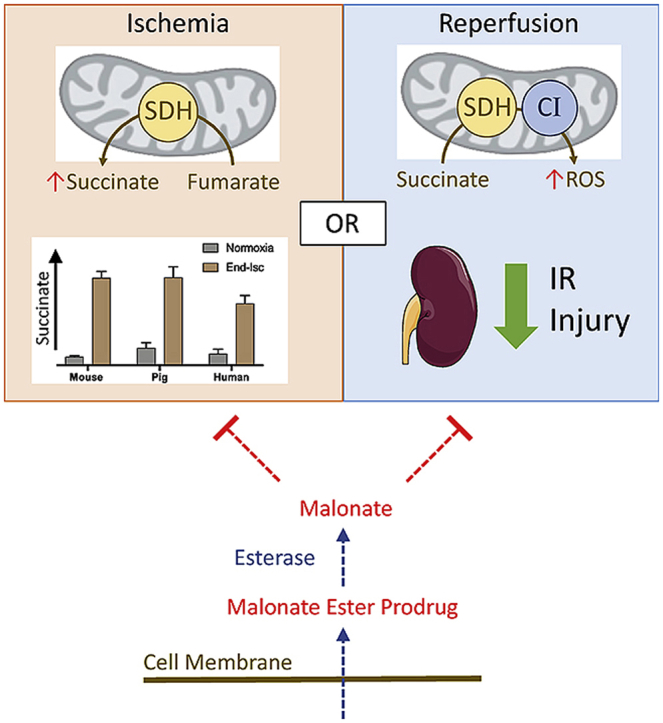

## 1 Introduction

Ischemia reperfusion (IR) injury occurs upon the return of an oxygenated blood supply to an organ or tissue following a period of ischemia [1,2]. Paradoxically, the return of oxygenated blood leads to an increase in the level of injury over and above that which occurs during ischemia alone [1,3]. This is due to the production of reactive oxygen species (ROS) during reperfusion, primarily from mitochondria, which go on to initiate much of the downstream damage leading to organ or tissue injury [4,5]. The kidney has the second highest mitochondrial content of any organ after the heart [6]. The majority of mitochondria are located within the proximal tubule cells of the kidney where they provide energy for the reabsorption of nutrients and ions from the filtrate and are the renal cell type most susceptible to IR injury [7]. The kidney undergoes IR injury in a number of clinical contexts including hypovolaemic shock [8], cardiothoracic surgery [9], transplantation [10] and the resection of renal tumours [11], which is associated with significant morbidity and mortality [12]. A number of promising interventions targeting mitochondria have previously been identified in pre-clinical models of renal IR injury [[13], [14], [15]]. Even so, none have so far been successfully translated to humans. This may partly reflect the greater complexity of injury in the clinical scenario, and amelioration of renal IR injury remains an urgent unmet need.

While the production of ROS on reperfusion was traditionally thought to occur at random sites within mitochondria as a result of damage to the electron transport chain, a specific mechanism of mitochondrial ROS production during IR injury has recently been proposed [16,17]. Succinate accumulates during ischemia due to reduction of the coenzyme Q (CoQ) pool that blocks succinate oxidation and which can also cause the reduction of fumarate to succinate by reversal of succinate dehydrogenase (SDH). Upon reperfusion the accumulated succinate is rapidly oxidised, driving ROS production by reverse electron transport (RET) at Complex I. The ROS production associated with RET at Complex I is driven by the near maximal proton motive force which develops upon reperfusion, due to the degradation of adenine nucleotides during ischemia that deprives the F_O_F_1_-ATP synthase of its substrate, in conjunction with a reduced CoQ pool. The superoxide produced by Complex I upon reperfusion is converted to hydrogen peroxide by manganese superoxide dismutase (MnSOD) and goes on to initiate much of the downstream damage leading to IR injury [2,18]. The accumulation of succinate during ischemia and its subsequent oxidation upon reperfusion has previously been demonstrated in the mouse kidney [19], however uncertainty remains as to whether the same mechanism occurs in human kidneys [20] and whether targeting SDH upon reperfusion has therapeutic potential.

**Fig. 1:**
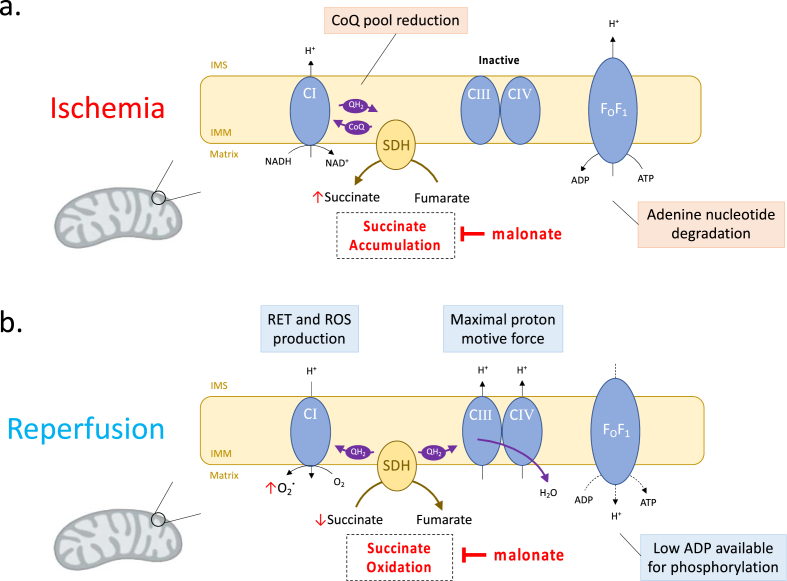
**Inhibition of Succinate Dehydrogenase During Ischemia Reperfusion Injury with Malonate.** (a) During ischemia, succinate accumulates due to reversal of succinate dehydrogenase (SDH). The accumulated succinate acts as a store of electrons capable of driving reverse electron transport (RET) and reactive oxygen species (ROS) production when rapidly re-oxidised on reperfusion. SDH reversal and succinate accumulation may be inhibited during ischemia by the competitive inhibitor of SDH, malonate. (b) On reperfusion, accumulated succinate is rapidly re-oxidised by SDH leading to reverse electron transport (RET) and the production of superoxide (O_2_**˙**) from the flavin mononucleotide site of Complex I (CI). Rapid re-oxidation of succinate by SDH on reperfusion may also be inhibited by the competitive inhibitor of SDH, malonate, which prevents RET and ROS production. Intermembrane space (IMS). Inner mitochondrial membrane (IMM).

**Fig. 2:**
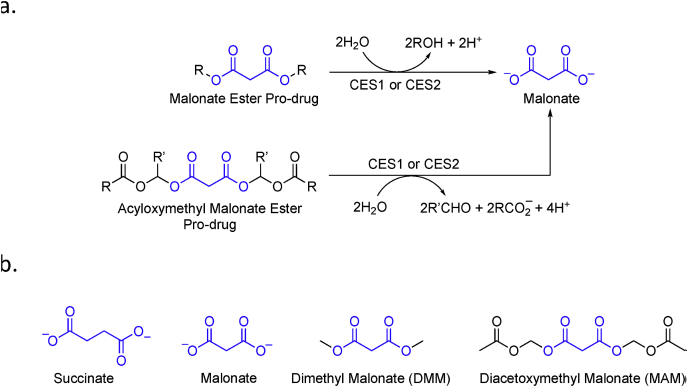
**Malonate Ester Prodrugs Used to Target Succinate Dehydrogenase *In Vivo*.** (a) Malonate ester prodrugs and particularly acyloxymethyl ester prodrugs are hydrolysed by intracellular carboxylesterases (CES1 and CES2) to release malonate. (b) Chemical structure of succinate, malonate, dimethyl malonate (DMM) and diacetoxymethyl malonate (MAM).

Malonate is a competitive inhibitor of SDH that can inhibit succinate accumulation during ischemia and its oxidation upon reperfusion, thereby reducing mitochondrial ROS production and IR injury (Fig. 1). However, malonate is a charged molecule and must be given as a cell-permeable prodrug to enter tissues *in vivo.* This can be achieved by administering malonate as an ester prodrug where the charged carboxylate groups are masked by short-chain alcohols, enabling the prodrug to passively diffuse through lipid membranes [21]. Once inside, intracellular carboxylesterases, such as CES1 and CES2, can hydrolyse the ester bonds of the prodrug, releasing the active drug malonate (Fig. 2a). Malonate ester prodrugs that are slowly hydrolysed by carboxylesterases may only be suited to inhibiting succinate accumulation during ischemia, while malonate ester prodrugs hydrolysed more rapidly may be suitable for the inhibition of succinate oxidation upon reperfusion as well [22]. Acyloxymethyl esters of carboxylic acids, carbamic acids and phosphonic and phosphoric acids are widely used as prodrugs because the acyl group is easily hydrolysed by carboxylesterases and tuneable [21]. This makes them particularly attractive as ester prodrugs of malonate (Fig. 2a).

Here, we first investigate the metabolic changes that occur during ischemia in mouse, pig and human kidneys to determine whether the mechanism of renal IR injury previously described in the mouse is conserved across species. We then investigate the efficacy of two malonate ester prodrugs, dimethyl malonate (DMM) and diacetoxymethyl malonate (MAM, an acyloxymethyl diester) (Fig. 2b), in targeting succinate metabolism during renal IR injury in the mouse. Acetoxymethyl groups are known to be rapidly hydrolysed by esterases and thus may result in more rapid production of therapeutic levels of malonate upon reperfusion [23]. We show that administration of DMM reduces mouse renal IR injury, but that diacetoxymethyl malonate, a more rapidly hydrolysed malonate prodrug, is far more potent at preventing kidney IR injury.

## 2 Materials and methods

### 2.1 Synthesis of diacetoxymethyl malonate

Diacetoxymethyl malonate (MAM) was prepared by O-alkylation of malonate with acetoxymethyl bromide following the procedure of Bao et al. [24].

### 2.2 Model of renal IR injury in the mouse

Female C57BL/6J mice, aged 10–16 weeks and weighing ~20g (Charles River Laboratories, UK) were maintained in specific pathogen-free facilities with *ad libitum* food and water. All procedures were approved by the UK Home Office under the Animals (Scientific Procedures) Act 1986. Mice were anaesthetised with 1.5–2% isoflurane and 2.0 L/min oxygen and their core temperature maintained at 36.0 ± 0.5 °C throughout the procedure using a homeothermic heat mat (Harvard Apparatus, UK). A midline laparotomy was performed and the renal hila were dissected. To induce ischemia, a Micro-Serrofine clamp (15 mm, Fine Science Tools, Germany) was placed over the renal hilum of each kidney to occlude the renal artery and vein. During ischemia, the midline laparotomy was closed using 5-0 absorbable suture (Safil®, Braun, Germany) to maintain core body temperature and minimise losses from evaporation. Successful occlusion of the renal vessels was confirmed by the development of a purplish colour to each kidney. For reperfusion experiments, Micro-Serrefine clamps were removed from the renal hila following ischemia and return of the kidney(s) to normal colour was noted.

In non-terminal procedures, the abdominal wall was closed in two layers with 5-0 absorbable suture (Safil®, Braun, Germany) and mice were re-hydrated with 500 μL saline injected subcutaneously. Mice were then recovered in an incubator set at 28 °C on soft, dry bedding with access to food and water. A subcutaneous injection buprenorphine (0.1 mg/kg) was given for pain relief in recovery experiments.

### 2.3 Infusions

In all experiments, compounds were administered as an infusion in a total volume of 160 μL saline. A syringe driver (Model 11 Syringe Pump, Harvard Apparatus) was used to deliver compounds at a rate of 16 μL/min through an infusion line (Portex Polyethylene Tubing, Smiths Medical International, UK) inserted directly into the inferior vena cava (IVC) with a 30 G needle. In experiments aimed at inhibiting succinate accumulation, saline or DMM (40, 80, 160, 320 and 640 mg/kg) were administered as an infusion beginning 10 min prior to the onset of ischemia. In experiments aimed at inhibiting succinate oxidation on reperfusion, saline, DMM (160 mg/kg), 1% v/v dimethyl sulfoxide (DMSO) or MAM (16 mg/kg) in 1% v/v DMSO were administered as a 10 min infusion beginning 5 min prior to the onset of reperfusion.

### 2.4 Administration of MitoB

MitoB and internal standards were synthesised as previously described [25]. MitoB (20 nmol in 50 μL saline + 5% (v/v) DMSO) was given as an intravenous injection directly into the IVC 30 min prior to the onset of ischemia.

### 2.5 Tissue collection and storage

Tissue and whole blood were collected from mice under terminal anaesthesia. Approximately 500 μL whole blood was collected directly from the IVC using a 1 mL syringe and 27 G needle. Whole blood was transferred to 250 μL capillary action collection tubes and allowed to clot. Tubes were then centrifuged at 3000 g for 10 min to separate the clot from the serum. The serum was then collected and stored at −70 °C until further processing and analysis. Kidneys for metabolomic analysis were rapidly excised from the mouse and frozen in liquid nitrogen (LN_2_) using Wollenburger clamps. Frozen tissue was then stored at −70 °C until further processing. Rapid removal and freezing of tissue in Wollenberger clamps was essential in preserving the metabolic profile of the kidney at each stage of the experiment [26].

### 2.6 Pig model of renal ischemia

Large male and female Landrace pigs weighing 40–70 kg were supplied by Convance, Huntington, UK. All procedures approved by the UK Home Office under the Animals (Scientific Procedures) Act 1986. Pigs were medicated with intramuscular ketamine (10 mg/kg), medetomidine (0.02 mg/kg) and midazolam (0.1 mg/kg). A peripheral intravenous catheter was placed in the marginal ear vein and anaesthesia was induced with propofol to effect. Pigs were intubated and 100% oxygen supplied with intermittent positive pressure ventilation was used to maintain normocapnia. Anaesthesia was maintained with continuous infusions of propofol (starting at 10 mg/kg/hr and titrating down to effect) and either remifentanil (starting at 2.4 mg/kg/h and titrating up to effect) or alfentanil (starting at 30 μg/kg/h and titrating up to effect). If required, isoflurane was provided at approximately 2% to maintain anaesthesia. Saline was administered intravenously at approximately 10 mL/kg/h. During anaesthesia a Datex Ohmeda Cardiocap monitoring system was used to monitor ECG waveform, pulse oximetry, temperature and capnography parameters.

A midline laparotomy was performed and the bowel mobilised to expose the renal hila. The renal hila were dissected and renal artery and vein slung using a 3-0 vicryl tie (Ethicon, Johnson & Johnson, UK) on both sides. The ureters were identified and each kidney was freed from the underlying connective tissue. Heparin (500 IU/kg, CP Pharmaceuticals, UK) was then administered via the marginal ear vein. Five minutes after the administration of heparin, a wedge tissue biopsy was taken from the kidney and rapidly clamp frozen in LN_2_ using Wollenburger clamps. The renal artery and vein of each kidney were then tied and the renal vessels and ureter divided. Kidneys were then maintained at the physiological temperature of 38 °C in the abdomen of the pig for 30 min [27]. At the end of 30 min ischemia, a second wedge biopsy was taken from each kidney and rapidly clamp frozen in LN_2_ using Wollenburger clamps. Frozen tissue was then stored at −70 °C until further processing.

### 2.7 Acceptance of declined human kidneys for research

Human kidneys declined for organ transplantation were accepted for research according to NHS Blood and Transplant guidelines. Full ethical approval for the use of human kidneys for the investigation of ischemia reperfusion injury had been awarded in advance (NREC 15/10.13039/100006147NE/0408) and informed consent from the families of organ donors was obtained. Declined human kidneys were delivered to our laboratory under conditions of static cold storage. Deceased donor demographics of declined human kidneys accepted for research are shown in Table 1.

**Table 1:** Deceased donor demographics of declined human kidneys used to investigate the metabolic changes in the kidney under conditions of warm ischemia.

Variables	Donor 1	Donor 2	Donor 3	Donor 4
Donor age (y)	55	65	64	69
Donor type	DCD^a^	DBD^b^	DCD	DCD
Reason for decline	Vascular damage	Vessel Anatomy	Vascular disease	Lesion on caecum
Static cold storage time (h)	17	6	13	12

a Donation after circulatory death.

b Donation after brainstem death.

### 2.8 *Ex vivo* normothermic perfusion of human kidneys

*Ex vivo* normothermic perfusion of human kidneys was conducted as previously described by Nicholson et al. [28]. In brief, declined human kidneys were reperfused for 1 h on a modified paediatric cardiopulmonary bypass circuit (Medtronic, UK) using a warmed (37.0 ± 1.0 °C), oxygenated, ABO-matched, whole-blood perfusate (300 mL whole blood, 300 mL Ringers solution (Baxter Healthcare, US), 2.5 g mannitol, 0.06 g creatinine, 10 mL 8.4% sodium bicarbonate, 500 IU heparin) at a constant pump-speed of 1500 rpm. During EVNP, Ringer's solution was used to replace the volume of perfusate lost in the urine (titrated to the rate of urine production) and the perfusate was supplemented with a 5% glucose solution (Baxter Healthcare, US) at a rate of 5 mL/h and an amino acid solution (Synthamin 17 10.0%, Baxter Healthcare, US) with added insulin (0.2 IU/mL) and 8.4% sodium bicarbonate (0.3 mL per mL Synthamin) at a rate of 20 mL/h.

Following 1 h EVNP, a tissue wedge biopsy was taken from cortex of the kidney and rapidly clamp frozen in LN_2_ using Wollenburger clamps. Declined human kidneys were then removed from the circuit, placed in a plastic bag and submerged in a water bath set to 36 °C to simulate renal warm ischemia. Following 30 min ischemia a second wedge biopsy was taken from the cortex of the kidney and rapidly clamp frozen in LN_2_ using Wollenburger clamps. Frozen tissue was then stored at −70 °C until further processing.

### 2.9 Extraction of polar metabolites for liquid chromotography tandem mass spectrometry (LC-MS/MS)

For each sample, approximately 20 mg of clamp frozen tissue was weighed out on dry ice into a pre-cooled Precellys tube (Hard tissue homogenising CK28-R - 2 mL; Bertin Instruments, France). After weighing, 25 μL/mg of pre-cooled extraction buffer (50% (v/v) mass spectrometry (MS)-grade methanol (Thermo Fisher Scientific, UK), 30% (v/v) MS-grade acetonitrile (Romil, UK), 20% MS-grade water (Thermo Fisher Scientific, UK) and 100 ng/mL HEPES free acid) was added to each sample along with 20 μL internal standard containing 1 nmol ^13^C_4_-succinate and 1 nmol ^13^C_3_-malonate. Samples were homogenised using a Precellys 24 tissue homogeniser (6500 rpm, 2 × 15 s, Bertin Instruments, France) and transferred back onto dry ice. Following homogenisation, samples were centrifuged (17,000 g, 10 min, 4 °C) and the supernatant transferred to a pre-cooled microcentrifuge tube on wet ice. Samples were centrifuged again (17,000 g, 10 min, 4 °C) and the supernatant transferred to a pre-cooled MS vial. Vials were stored at −70 °C until analysis.

### 2.10 Extraction of MitoP and MitoB for LC-MS/MS

For each sample, approximately 50 mg of clamp frozen tissue was weighed out on dry ice into a 2 mL microcentrifuge tube. One spatula of zirconium oxide beads (Next Advance, USA), approximately the size of the tissue, was added to each sample along with 200 μL MitoP/B extraction buffer A (60% (v/v) MS-grade acetonitrile (Romil, UK), 0.1% MS-grade formic acid (Merck, UK), 39.9% MS-grade water (Thermo Fisher Scientific, UK)) and 10 μL internal standard containing 100 pmol *d*_*15*_-MitoB and 50 pmol *d*_*15*_-MitoP. Samples were then homogenised using a Bullet Blender (speed 8, 4 min, Next Advance, USA) and left for 30 min at 4 °C. Samples were then centrifuged (17,000 g, 10 min) and the supernatant transferred to a 96 well filter plate. The sample pellet was then resuspended in 200 μL MitoP/B extraction buffer A, vortexed for approximately 30 s and centrifuged again (17,000 g, 10 min). The second supernatant was then added to first supernatant in the corresponding well of the filter plate and samples were vacuum filtered into the collection plate. The filtered supernatant of each sample was then transferred to a fresh 2 mL microcentrifuge tubes and dried in a speed vac overnight (40 °C). Dried samples were then resuspended in 250 μL MitoP/B extraction buffer B (20% (v/v) MS-grade acetonitrile (Romil, UK), 0.1% MS-grade formic acid (Merck, UK), 79.9% MS-grade water (Thermo Fisher Scientific, UK)), vortexed for 5 min and centrifuged (17,000 g, 10 min). The supernatant was then transferred to MS vials and stored at −70 °C until analysis.

### 2.11 LC-MS/MS of succinate and malonate

Samples were analysed using an LCMS-8060 mass spectrometer (Shimadzu, UK) with a Nexera *X*2 UHPLC system (Shimadzu, UK). Sample separation was achieved using a SeQuant®ZIC®-HILIC column (3.5 μg, 100 Å, 150 × 2.1 mm, 30 °C column temperature; MerckMillipore, UK) with a ZIC®-HILIC guard column (200 Å, 1 × 5 mm). A flow rate of 200 μl/min was used with mobile phases of 10 mM ammonium bicarbonate (pH unchanged) and B) 100% acetonitrile. The mass spectrometer was operated in negative ion mode with multiple reaction monitoring (MRM). Sample spectra were acquired using Labsolutions software (Shimadzu, UK) and the peak area for each compound of interest measured relative to the internal standard. Sample concentrations were then calculated from a standard curve of known compound concentrations produced by LC-MS/MS in a similar manner.

### 2.12 LC-MS/MS of MitoP and MitoB

A Waters Xevo TQ-S triple-quadrupole mass spectrometer was used for MS analysis. Electrospray ionization was in positive ion mode; parameters were as follows: capillary voltage, 3.2 kV; cone voltage, 79 V; ion source temperature, 150 °C; collision energy, 45 V. Nitrogen and argon were used as the curtain and the collision gases, respectively. LC-MS/MS analyses were carried out using a front end I-class Aquity LC system (Waters). Samples were cooled and 2 mL injected using a 15 mL flow-through needle and RP-HPLC was carried out at 30 °C using an Acquity UPLC BEH C18 1.μm, 1 3 50 mm column (Waters). Buffers were 5% (v/v) ACN/0.1% (v/v) formic acid (FA) in water (MS buffer A) and 90% (v/v) ACN/0.1% (v/v) FA (MS buffer B). A gradient was run at 200 mL/min: 0–0.3 min, 5% B; 0.3–3 min, 5–100% B; 4–4.1 min, 100–5% B; 4.1–4.6 min, 5% B. Eluent was diverted to waste at 0–1 and 4-4.6 min acquisition time using an in-line divert valve. Multiple reaction monitoring in positive ion mode was used for compound detection.

### 2.13 ATP/ADP luciferase assay

ATP and ADP concentrations were measured using a Luciferase based assay as described by Strehler et al. [29] and performed by Martin et al. [30]. In brief, approximately 10 mg of clamp frozen tissue was weighed out on dry ice into pre-cooled Precellys tubes (Hard tissue homogenising CK28-R - 2 mL; Bertin Instruments, France). After weighing, 100 μL/mg of ice-cold perchloric acid extractant (3% (v/v) HClO_4_, 2 mM Na_2_-EDTA, 0.5% Triton X-100) was added to each sample. Samples were then homogenised using a Precellys 24 tissue homogeniser (6500 rpm, 2 × 15 s, Bertin Instruments, France) transferring samples back onto wet ice between cycles. Following homogenisation, samples were centrifuged (10,000g, 1 min, 4 °C) and the supernatant diluted in ice cold perchloric acid extractant to a concentration of 1 mg frozen tissue per mL. Samples along with ATP and ADP standards were neutralised to pH 7 using potassium hydroxide solution (2 M KOH, 2 mM Na_2_-EDTA, 50 mM MOPS). To measure ADP concentration, 250 μl of neutralised sample was added to 250 μL ATP sulfurylase assay buffer (20 mM Na_2_MoO_4_, 5 mM GMP, 0.2 U ATP sulfurylase (New England Biolabs, USA), in Tris-HCl buffer (100 mM Tris-HCl, 10 mM MgCl_2_ (pH 8.0)) and incubated for 30 min at 30 °C to convert the sample's ATP to AMP. ADP samples were then heated at 100 °C for 5 min and then cooled on ice. Standards (100 μL), samples for ATP measurement (100 μL) or samples for ADP measurement (200 μL) (in duplicate) were then added to 400 μL Tris-acetate (TA) buffer (100 mM Tris, 2 mM Na_2_-EDTA, 50 mM MgCl_2_, pH 7.75 with glacial acetic acid) in luminometer tubes. To measure ADP concentration, 10 μL pyruvate kinase solution (100 mM PEP, 6 U pyruvate kinase suspension (Sigma #P1506)) was added to one set of samples for ADP measurement and incubated for 30 min at 25 °C in the dark to convert ADP to ATP. The other duplicate tube (without addition of pyruvate kinase solution) served as an ADP ‘blank’ value. The samples were then all assayed for ATP content in a Berthold AutoLumat Plus luminometer by addition of (100 μL) Luciferase/Luciferin Solution (7.5 mM DTT, 0.4 mg/mLBSA, (1.92 μg) luciferase/mL (SIGMA #L9506), 120 μM D-luciferin (SIGMA #L9504), made in TA buffer (25% (v/v) glycerol)), delivered via auto injection, protected from light. Bioluminescence of the ATP- dependent luciferase activity was measured for 45 s post injection and the data quantified against standard curves.

### 2.14 Measurement of serum creatinine

Serum creatinine was measured on an automated biochemical analyser (Siemens Dimension RxL analyser, Siemens AG, Healthcare Division, Germany) by the Jaffe reaction. This assay was performed by Cambridge Biochemical Analysis Laboratory, Addenbrooke's Hospital, Cambridge, UK.

### 2.15 Statistical analysis

Mice were picked at random from the same cage and assigned to control or experimental arms. Equal numbers of right and left kidneys were allocated to each experimental group. MS and serum creatinine samples were analysed blinded to the experimental group identity. Data are presented as mean ± SEM. Statistical analysis was performed using Prism 7.0 (Graphpad, USA). Comparisons between two datasets were assessed using an unpaired, two-tailed Student's t-test assuming equal variance. We note that due to the number of data points it was not possible to confirm normality of distribution in all cases. Comparisons between multiple datasets were assessed using one or two-way analysis of variance (ANOVA) followed by Dunnett's, Tukey's or Sidak's multiple comparisons test. P values < 0.05 were considered to be statistically significant.

## 3 Results

### 3.1 Ischemia time and injury severity following renal IR in the mouse

**Fig. 3:**
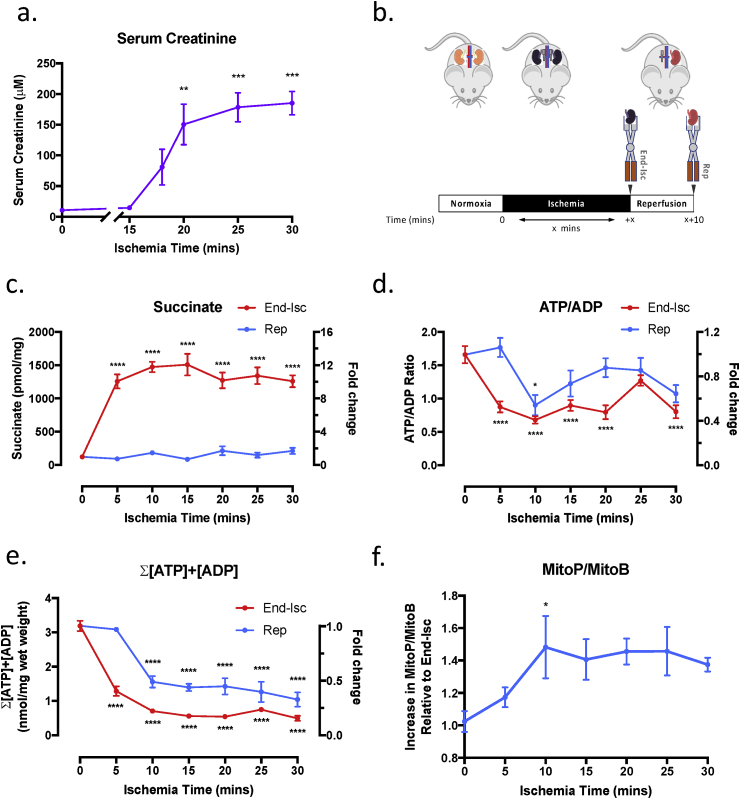
**Metabolic Changes During Ischemia Reperfusion in the Mouse Kidney.** (a) Serum creatinine concentration (n = 3–4) at 24 h reperfusion following bilateral renal ischemia for the indicated durations. Control values represent serum creatinine concentration in age-matched mice that have not undergone bilateral renal IR injury. (b) Model used to investigate the metabolic changes during renal IR injury in the mouse. Mice underwent bilateral renal ischemia for the indicated durations, at the end of which one kidney was removed and rapidly clamp frozen in LN_2_ using Wollenburger clamps (End-Isc). The second kidney was reperfused for 10 min and then removed and rapidly clamp frozen in LN_2_ using Wollenberger clamps also (Rep). (c) Tissue succinate concentration (n = 4), (d) ATP/ADP ratio (n = 3–4) and (e) sum of the ATP and ADP concentrations (n = 3–4) at the end of ischemia (End-Isc) and at 10 min reperfusion (Rep). Control kidneys (0 min) were removed from the mouse under conditions of normoxia. (f) Relative increase in MitoP/MitoB ratio on reperfusion (n = 3–4) calculated from the MitoP/MitoB ratio in the kidney at 10 min reperfusion (Rep) over the MitoP/MitoB ratio at the end of ischemia (End-Isc). *P < 0.05, **P < 0.01, ***P < 0.001, ****P < 0.0001. P values were calculated by one-way ANOVA with Dunnett's multiple comparison test. Data are mean ± SEM.

In order to investigate the therapeutic effect of malonate ester prodrugs on renal IR injury we first established a mouse model. To do this we investigated varying periods of bilateral renal ischemia followed by reperfusion (of both kidneys) with the level of serum creatinine concentration at 24 h used to measure the extent of renal IR injury (Fig. 3a). Exposure to 15 min ischemia had no effect on serum creatinine (Fig. 3a), likely due to the large reserve capacity of the kidney, while mice exposed to more than 30 min ischemia did not survive reperfusion. Intermediate levels of ischemia (18–30 min) led to a significant increase in creatinine at 24 h with longer ischemia times leading to prolonged recovery of animals on reperfusion.

### 3.2 Succinate metabolism during renal IR injury in the mouse

We next investigated the metabolic changes in the rapidly frozen [26] mouse kidney at the end of various periods of ischemia and also following 10 min reperfusion after ischemia (Fig. 3b). Tissue succinate levels were increased ~10 fold compared to normoxic controls at all ischemic times and reached a maximum within 5 min of ischemia (Fig. 3c). After 10 min reperfusion following all periods of ischemia, the tissue succinate level returned to normoxic levels. Thus succinate is accumulated during ischemia and rapidly oxidised upon reperfusion in our model of kidney IR injury.

### 3.3 Changes in ATP and ADP during renal IR injury in the mouse

We next determined the ATP/ADP ratio and the total ATP and ADP concentration within the kidney during ischemia and upon reperfusion. The ATP/ADP ratio decreased within 5 min ischemia and remained low (Fig. 3d). The ATP/ADP ratio recovered fully after 10 min reperfusion following 5 min ischemia, but the recovery was less following longer periods of ischemia (Fig. 3d).

The sum of the concentration of the adenine nucleotides, ATP and ADP, decreased at all times of ischemia (Fig. 3e). Upon 10 min reperfusion after various times of ischemia, the sum of the ATP and ADP concentrations recovered fully after 5 min ischemia, but remained significantly decreased following longer ischemia times. This suggests ATP could be rapidly regenerated from ADP upon reperfusion following 5 min ischemia but not at longer ischemic times [19].

### 3.4 Mitochondrial ROS production during renal IR injury in the mouse

To measure mitochondrial ROS production in the kidney upon reperfusion, mice were administered the ratiometric MS probe, MitoB [31] which reacts selectively with hydrogen peroxide within mitochondria to form MitoP. There was a significant increase in the MitoP/MitoB ratio upon reperfusion following 10 min ischemia (Fig. 3f) suggesting a burst of mitochondrial ROS production had occurred.

### 3.5 Conservation of metabolic changes during ischemia in mouse, pig and human kidneys

**Fig. 4:**
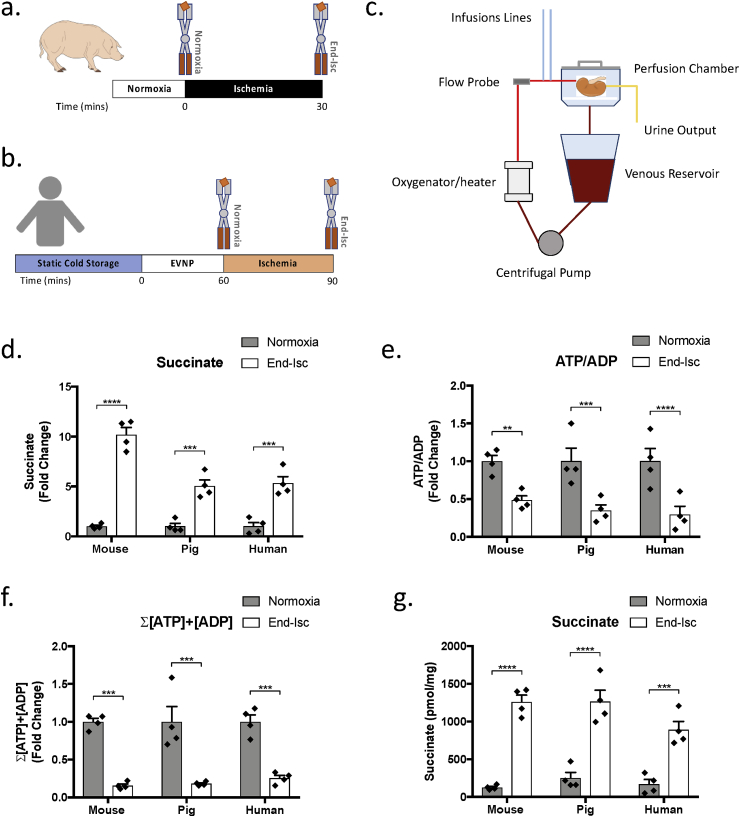
**Metabolic Changes in Mouse, Pig and Humans During Renal Ischemia**. (a) A tissue wedge biopsy was taken from the kidney of anaesthetised pigs under conditions of normoxia and rapidly clamp frozen in LN_2_ using Wollenburger clamps. The renal vessels were then tied and divided, and the pig kidney was exposed to 30 min ischemia in the abdomen of the pig maintained at the physiological of temperature 38 °C [27]. A second wedge biopsy was taken at the end of 30 min ischemia. (b) Human kidneys retrieved for transplantation but subsequently offered for research were reperfused with oxygenated ABO-matched blood for 1 h using *ex vivo* normothermic perfusion (EVNP). A wedge biopsy was taken from the kidney at the end of 1 h to act as a normoxic control. Human kidneys were then exposed to 30 min ischemia at a temperature of 36 °C. At the end of 30 min ischemia, a second wedge biopsy was taken. (c) Schematic of the EVNP circuit. (d) Succinate concentration (n = 4), (e) ATP/ADP ratio (n = 4) and (f) sum of the ATP and ADP concentration (n = 4) in the kidneys of mice, pigs and humans following 30 min ischemia (End-Isc) relative to normoxia. (g) Absolute tissue succinate concentration in the kidneys of mice, pigs and humans during normoxia and following 30 min ischemia (End-Isc). Mouse kidneys were exposed to 30 min ischemia as previously described in Fig. 3b. **P < 0.01, ***P < 0.001, ****P < 0.0001. P values were calculated by two-way ANOVA with Sidak's multiple comparisons test. Data are mean ± SEM.

To determine whether our findings in the mouse were translatable, we next investigated the metabolic changes that occur in the pig and human kidney during ischemia. The kidneys were removed from anesthetised pigs and exposed to 30 min ischemia (Fig. 4a). Human kidneys retrieved for transplantation but subsequently offered for research were first reperfused for 60 min with oxygenated blood using an *ex vivo* normothermic perfusion (EVNP) system, then exposed to 30 min ischemia (Fig. 4b and c) [28,32]. During EVNP, declined human kidneys had an average (mean ± SD, N = 4) renal blood flow of 83 ± 32 mL/min/100g and a total urine output of 12 ± 11 mL/h/100g.

In both pig and human kidneys 30 min ischemia led to a 5-fold increase in succinate (Fig. 4d), a decrease in the ATP/ADP ratio (Fig. 4e) and a drop in the total ATP and ADP nucleotide concentrations (Fig. 4f) compared to normoxia. Of note, the absolute levels of succinate that accumulated after ischemia were similar across the three species (Fig. 4g). Together these findings suggest that ischemic succinate metabolism in the kidney is conserved in mice, pigs and humans.

### 3.6 Administration of DMM prior to ischemia is not protective against renal IRI

**Fig. 5:**
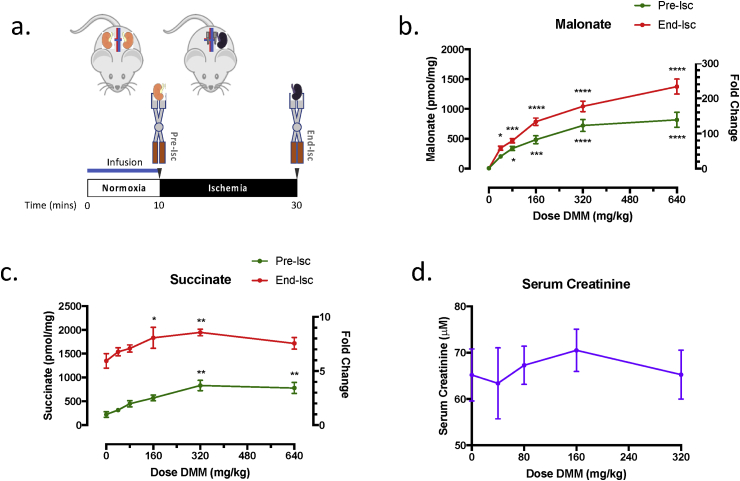
**Targeting Succinate Accumulation with Dimethyl Malonate during Ischemia in the Mouse Kidney.** (a) DMM was administered at the indicated doses in 160 μL saline as an infusion (rate 16 μL/min) 10 min prior to the onset of ischemia. At the end of the infusion, immediately prior to ischemia, one kidney was removed and rapidly clamp frozen in LN_2_ using Wollenberger clamps (Pre-Isc). The second kidney was exposed to 20 min warm ischemia and then rapidly clamp frozen (End-Isc). Control kidneys were given a 160 μL infusion of saline only. (b & c) Tissue malonate (n = 4) and succinate (n = 4) concentration at the end of the infusion (Pre-Isc) and following 20 min ischemia (End-Isc). (d) Serum creatinine concentration (n = 4) at 24 h reperfusion following infusion of saline or DMM prior to ischemia as described in (a) followed by 20 min bilateral renal ischemia. *P < 0.05, **P < 0.01, ***P < 0.001, ****P < 0.0001. P values were calculated by two-way ANOVA with Sidak's multiple comparison test (b & c) and one-way ANOVA with Dunnett's multiple comparison test (d). Data are mean ± SEM. Of note, 640 mg/kg DMM administered prior to ischemia was toxic to the mice and the serum creatinine concentration at 24 h reperfusion could not be measured.

To determine whether malonate ester-prodrugs inhibit succinate accumulation during ischemia in the mouse kidney, DMM was given as an infusion to mice 10 min prior to the onset of ischemia (Fig. 5a). There was a dose-dependent increase in the malonate concentration in DMM-treated mice at the end of the infusion and after 20 min ischemia (Fig. 5b). However, there was no decrease in the succinate concentration by malonate at 20 min ischemia (Fig. 5c). There was also an increase in succinate at higher DMM concentrations prior to ischemia due to the inhibition of SDH during normoxia (Fig. 5c). Furthermore, the serum creatinine concentration after 24 h reperfusion was not reduced by DMM (Fig. 5d). Therefore administration of DMM prior to ischemia is not protective against renal IR injury.

### 3.7 Administration of malonate esters upon reperfusion is protective against renal IRI

**Fig. 6:**
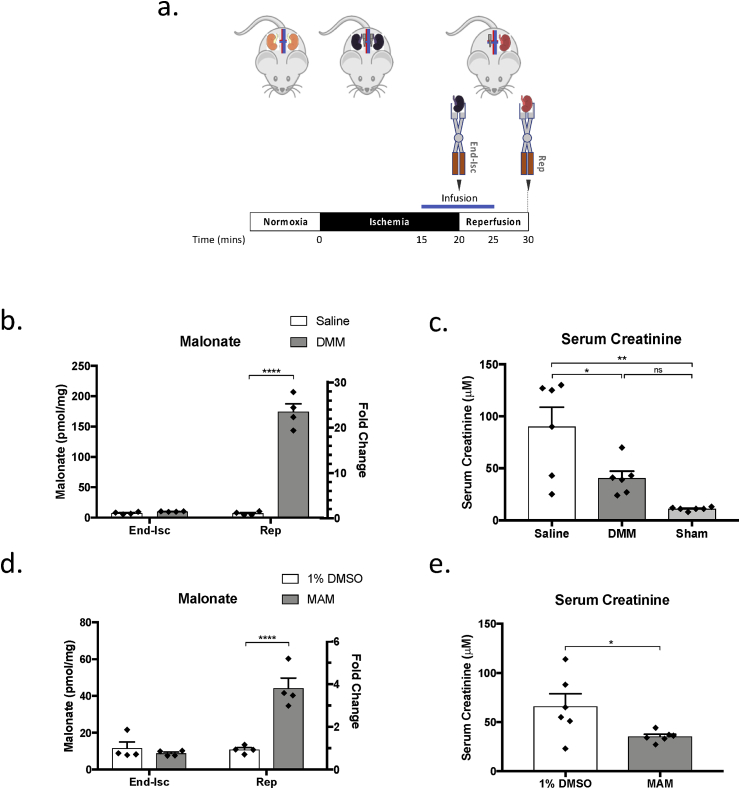
**Targeting Succinate Oxidation on Reperfusion with Dimethyl Malonate and Diacetoxymethyl Malonate in the Mouse Kidney.** (a) Mice underwent 20 min bilateral renal ischemia. At the end of ischemia, one kidney was removed and rapidly clamp frozen in LN_2_ using Wollenburger clamps (End-Isc). The second kidney was reperfused for 10 min and then removed and rapidly clamp frozen (Rep). An infusion of 160 μL 160 mg/kg dimethyl malonate (DMM) or 16 mg/kg diacetoxymethyl malonate in 1% DMSO (MAM) was given at a rate of 16 μL/min starting 5 min prior to the onset of reperfusion. Control kidneys were given an infusion of saline or 1% DMSO only. (b) Tissue malonate (n = 4) concentration at the end of 20 min ischemia (End-Isc) and at 10 min reperfusion (Rep) in mice infused with 160 mg/kg DMM. (c) Serum creatinine concentration (n = 6) at 24 h reperfusion following 20 min bilateral renal ischemia and infusion of saline or 160 mg/kg of DMM as described in (a). Sham mice were infused with 160 μL saline at a rate 16 μL/min but did not undergo bilateral renal ischemia. (d) Tissue malonate (n = 4) concentration at the end of 20 min ischemia (End-Isc) and at 10 min reperfusion (Rep) in mice infused with 16 mg/kg MAM. (e) Serum creatinine concentration (n = 6) at 24 h reperfusion following 20 min bilateral renal ischemia and infusion of 1% DMSO or MAM as described in (a). *P < 0.05, **P < 0.01, ****P < 0.0001. P values were calculated by two-way ANOVA with Sidak's multiple comparison test (b & d), one-way ANOVA with Tukey's multiple comparison test (c) and an unpaired, two-tailed Student's t-test assuming equal variance (e). Data are mean ± SEM.

We next investigated whether malonate ester prodrugs could inhibit succinate oxidation when administered upon reperfusion. DMM (160 mg/kg) was administered upon reperfusion of mice that had undergone 20 min bilateral renal ischemia (Fig. 6a). DMM-infusion increased the malonate concentration at 10 min reperfusion (Fig. 6b). Serum creatinine concentration measured 24 h after reperfusion was significantly decreased in DMM-treated mice compared to controls (Fig. 6c) suggesting DMM administered at reperfusion exerted a protective effect. It is probable that the protective effect of DMM was due to the inhibition of succinate oxidation by SDH on reperfusion as succinate was higher at the end of ischemia with 160 mg/kg DMM than in the absence of DMM (Fig. 5c) [33,34].

To improve the efficacy of malonate ester prodrug delivery, we also assessed the malonate ester prodrug diacetoxymethyl malonate (MAM), which is hydrolysed more rapidly than DMM thereby increasing the rate of malonate generation within the cell, thus allowing lower doses and/or better protection when given at reperfusion [23]. To test whether MAM was protective at lower doses than DMM, MAM was administered to mice at a 10x lower concentration than DMM following 20 min bilateral renal ischemia (Fig. 6a).

Malonate was significantly increased in the kidneys of MAM-treated mice compared with controls at 10 min reperfusion (Fig. 6d). The serum creatinine concentration was significantly decreased in MAM treated mice at 24 h, suggesting MAM had a protective effect (Fig. 6e). Again, this was likely due to the inhibition of succinate oxidation by SDH on reperfusion [33,34]. Importantly, MAM also resulted in a reduction in serum creatinine 24 h after reperfusion (Fig. 6e), but at a 10x lower concentration to DMM. The fine tuning of malonate ester prodrugs is therefore an attractive method of improving the efficacy of these compounds and reducing their unwanted off-target effects [22].

## 4 Discussion

In this study, we show that malonate ester prodrugs may ameliorate IR injury in the mouse kidney if administered upon reperfusion, but not if given prior to ischemia. We show similar metabolic changes occur during ischemia in the pig and human kidney highlighting the potential translatability of our findings in the mouse. Furthermore, we show that the ‘tuned’ malonate ester prodrug, MAM, may achieve the same protective effect as the simple malonate ester prodrug, DMM, at a 10x lower concentration. The faster hydrolysis of MAM and lower concentration required for therapeutic effect is important in the specific targeting of succinate oxidation by SDH on reperfusion and in reducing the off-target effects of these compounds [22]. Given the transient increase in tissue malonate concentration produced by the malonate ester-prodrugs administered on reperfusion [33,34], it is likely that the protective effects of DMM and MAM demonstrated in this study are due to the inhibition of succinate oxidation by SDH on reperfusion, leading to a reduction in mitochondrial ROS production. MitoB was not sensitive enough to detect a significant increase in mitochondrial ROS production on reperfusion in this model and so the effect of DMM and MAM could not be measured directly. Nevertheless, as malonate is readily metabolised and excreted, its protective effect is unlikely to result from longer-term SDH inhibition as has been shown previously [35].

Administration of DMM prior to ischemia in the mouse kidney did not lead to a reduction in the succinate concentration, despite apparent inhibition of SDH. This is in contrast to previous studies in the rat brain [19] and mouse heart [30] although differences in compound administration, ischemia times and temperatures may also play a role. The metabolic conditions required for succinate accumulation via SDH reversal are thought to include a highly reduced NAD^+^/NADH pool, and inactivity of complexes III and IV due to the lack of oxygen [16]. It is unlikely these conditions occur instantaneously on the onset of ischemia but develop over seconds to minutes, after which SDH reversal may begin. In the intervening period other pathways, such as glutaminolysis and canonical citric acid cycle (CAC) action, may contribute to succinate accumulation [36]. There is uncertainty as to the contribution of canonical CAC action to overall succinate accumulation and it may be that the contribution of different pathways varies between organs. Metabolic tracing experiments may allow this question to be addressed but were beyond the scope of our present study.

The time of administration of DMM appears to be critical in determining its efficacy. Whilst a high malonate concentration was achieved in the kidneys of mice treated with DMM prior to the onset of ischemia, this did not lead to a reduction in renal injury compared to mice treated with DMM at reperfusion. As malonate ester prodrugs are not specifically targeted to mitochondria and rely on passive diffusion into cells and hydrolysis by intracellular carboxylesterases in different cellular compartments [21], a number of factors may be at play that led to the differences in protection.

This is the first study to demonstrate succinate accumulation occurs in the human and pig kidney during ischemia. EVNP of human kidneys retrieved for transplantation but subsequently offered for research provided a unique opportunity to measure the concentration of succinate, ATP and ADP under conditions of normoxia. Although human kidneys retrieved for transplantation had previously been exposed to varying periods of static cold storage, succinate rapidly returns to normoxic levels on reperfusion and is unaffected by ischemic preconditioning [37]. Therefore this enabled us to take tissue biopsies from human kidneys during normoxia. This experimental platform also enabled human kidneys to be re-exposed to ischemia in a controlled manner, which is difficult to achieve in clinical settings.

The similar levels of succinate measured in the mouse, pig and human under conditions of normoxia and following 30 min ischemia strongly suggests a conserved mechanism of succinate accumulation in the human kidney following EVNP and re-exposure to ischemia. As such, EVNP may be used to measure the metabolic changes during kidney ischemia and offers an attractive platform to investigate the use of malonate ester prodrugs within the human kidney in future translational studies.

One limitation of this study is the reliance on serum creatinine as a sole marker of tissue injury. The mouse model of renal IR injury is known to be highly variable with the degree of injury affected by animal age, hydration status, core temperature, surgery time, surgical trauma, and post-operative care [38]. Numerous steps were taken to minimise the degree of variability as outlined in the Methods, including the use of a homeothermic heat mat to closely control the core temperature of the mouse during surgery and closure of the midline incision during ischemia to minimise fluid loss from evaporation. All surgeries were also performed by the same surgeon. However, an element of variability was unavoidable and may relate to changes in other factors such as surgical skill level over time. This may partly explain the variability in serum creatinine levels within this study, although all experiments consisted of contemporaneous control and treatment groups. Furthermore, a significant increase in tissue injury as measured by serum creatinine was only detected after 18 min ischemia. This is likely due to the large reserve capacity of the kidney with up to 50% of nephrons needing to be lost before a rise in serum creatinine concentration can be detected [39]. The findings of this study would therefore be strengthened by the analysis of other markers of tissue damage as well as histological analysis which would enable the protective effect of malonate esters on the different renal cell types to be probed further. In addition, experiments were performed using female mice only and should be confirmed in male mice thought to be more susceptible to IR injury [40].

## 5 Conclusion

In conclusion, we show that succinate driven RET and mitochondrial ROS production act as an important therapeutic target in renal IR injury. The rapid oxidation of succinate by SDH may be inhibited by the administration of malonate ester prodrugs on reperfusion, leading to a reduction in mitochondrial ROS production. The alcohol groups of malonate ester prodrugs may be fine-tuned to increase their rate of hydrolysis within tissue, enabling them to act more rapidly and reduce the overall concentration of prodrug required to achieve therapeutic effect, thereby reducing unwanted off-target effects. Whilst promising results have been demonstrated in the mouse, translational studies are now required to demonstrate similar protective effects in the pig and human before malonate ester prodrugs may be considered for early clinical trials.

## Funding

T.E.B was supported by a PhD studentship funded by the 10.13039/100010269Wellcome Trust Translational Medicines and Therapeutics Programme. Work in the M.P.M. laboratory was supported by the 10.13039/501100000265Medical Research Council UK (MC_U105663142) and by a 10.13039/100010269Wellcome Trust Investigator award (110159/Z/15/Z) to M.P.M. Work in the RCH lab laboratory was supported by a 10.13039/100010269Wellcome Trust Investigator award (110158/Z/15/Z) and a PhD studentship for L.P from the 10.13039/501100000853University of Glasgow. The human aspects of this research were funded by the 10.13039/501100000272National Institute for Health Research Blood and Transplant Research Unit (NIHR BTRU) in Organ Donation and Transplantation at the 10.13039/501100000735University of Cambridge in collaboration with 10.13039/501100000774Newcastle University and in partnership with 10.13039/100009033NHS Blood and Transplant (NHSBT). The views expressed are those of the author(s) and not necessarily those of the NIHR, the Department of Health and Social Care or NHSBT.

## Contributions

T.E.B, M.P.M and K·S-P designed the experimental protocols. T.E.B performed the experiments. MAM was synthesised and provided by L.P. Mass spectrometry was conducted by H.A.P and A.L. A.V.G assisted with ATP and ADP measurements. M.M.H and J.L.M assisted with mouse experiments. M.O.H, J.L.M and K.M assisted with pig experiments. M.L.N and S.A.H assisted with human perfusion experiments. T.E.B, M.P.M and K·S-P wrote the manuscript with assistance from all other authors.

## Declaration of competing interest

M.P.M. and K·S-P. have submitted a patent application on the use of dimethyl malonate to prevent IR injury.
